# Tris[(6*S*)-6-hy­droxy-4-*epi*-shikimic acid] monohydrate: an enanti­omerically pure hy­droxy­lated shikimic acid derived from methyl shikimate

**DOI:** 10.1107/S1600536812041256

**Published:** 2012-10-20

**Authors:** Axel G. Griesbeck, Claus Miara, Jörg-M. Neudörfl

**Affiliations:** a Department of Chemistry, University of Cologne, Greinstr. 4, 50939 Koeln, Germany

## Abstract

The title compound, 3C_7_H_10_O_6_·H_2_O, is the enanti­omerically pure product of a multi-step synthesis from the enanti­omerically pure natural shikimic acid. The asymmetric unit contains three mol­ecules of the acid and one mol­ecule of water. The cyclo­hexene rings of the acids have half-chair conformations. The carboxyl­ate, the four hydroxide groups and the additional water mol­ecule form a complex three-dimensional hydrogen-bonding network.

## Related literature

A series of anti­tumor-active marine natural carbasugars has been isolated in the last two decades with a cyclo­hexene-1-carboxyl­ate core structure and four contiguous stereogenic centers (Numata *et al.*, 1997 ▶). The relative configuration of these compounds, the pericosines, has been a matter of debate since the first reports on the isolation (Usami *et al.*, 2008 ▶, 2009 ▶). By means of detailed NMR analysis of the natural compound pericosine D0 and comparison with the NMR data published for the 6-hy­droxy-5-epishikimic acid described herein, the absolute and relative configuration was established (Usami *et al.*, 2006 ▶, 2011 ▶). This reveals the importance of this X-ray crystallographic determination that finally proves the assignments that resulted from spectroscopic analyses. For the synthesis, see: Griesbeck *et al.* (2007 ▶).
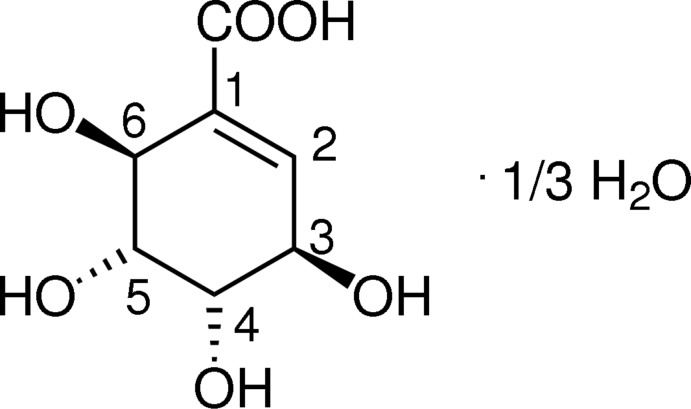


## Experimental

### 

#### Crystal data

3C_7_H_10_O_6_·H_2_O
*M* *_r_* = 588.47
Monoclinic, 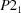

*a* = 11.2561 (17) Å
*b* = 7.7049 (11) Å
*c* = 13.9688 (14) Å
β = 91.672 (8)°
*V* = 1211.0 (3) Å^3^
*Z* = 2
Mo *K*α radiation
μ = 0.15 mm^−1^
*T* = 100 K
0.20 × 0.10 × 0.05 mm

#### Data collection

Nonius KappaCCD diffractometer
5629 measured reflections
2786 independent reflections
1399 reflections with *I* > 2σ(*I*)
*R*_int_ = 0.085

#### Refinement

*R*[*F* ^2^ > 2σ(*F* ^2^)] = 0.054
*wR*(*F* ^2^) = 0.102
*S* = 0.88
2786 reflections
371 parameters
3 restraints
H atoms treated by a mixture of independent and constrained refinement
Δρ_max_ = 0.27 e Å^−3^
Δρ_min_ = −0.28 e Å^−3^

### 

Data collection: *COLLECT* (Hooft 1998 ▶); cell refinement: *DENZO* (Otwinowski & Minor 1997 ▶); data reduction: *DENZO*; program(s) used to solve structure: *SHELXS97* (Sheldrick, 2008 ▶); program(s) used to refine structure: *SHELXL97* (Sheldrick, 2008 ▶); molecular graphics: *SCHAKAL99* (Keller 1999 ▶); software used to prepare material for publication: *PLATON* (Spek 2009 ▶).

## Supplementary Material

Crystal structure: contains datablock(s) global, I. DOI: 10.1107/S1600536812041256/gg2099sup1.cif

Supplementary material file. DOI: 10.1107/S1600536812041256/gg2099Isup2.cdx

Structure factors: contains datablock(s) I. DOI: 10.1107/S1600536812041256/gg2099Isup3.hkl

Additional supplementary materials:  crystallographic information; 3D view; checkCIF report

## Figures and Tables

**Table 1:** Hydrogen-bond geometry (Å, °)

*D*—H⋯*A*	*D*—H	H⋯*A*	*D*⋯*A*	*D*—H⋯*A*
O2—H2⋯O1*A* ^i^	0.84	1.81	2.624 (5)	164
O3—H3⋯O4*A* ^ii^	0.84	1.90	2.705 (5)	161
O4—H4⋯O6^iii^	0.84	1.94	2.738 (6)	157
O5—H5⋯O6*B* ^iv^	0.84	2.08	2.736 (5)	134
O6—H6⋯O3^iii^	0.84	1.89	2.718 (6)	169
O6*A*—H6*A*⋯O3*A* ^v^	0.84	1.99	2.764 (6)	153
O4*A*—H4*A*⋯O6*A* ^v^	0.84	1.88	2.712 (6)	168
O5*B*—H5*B*⋯O5	0.84	1.93	2.765 (5)	171
O5*A*—H5*A*⋯O3*B*	0.84	2.06	2.882 (5)	164
O2*A*—H2*A*⋯O1^vi^	0.84	1.84	2.664 (5)	167
O6*B*—H6*B*⋯O1*B* ^iv^	0.84	2.05	2.801 (5)	149
O4*B*—H4*B*⋯O4*A*	0.84	2.10	2.835 (6)	147
O2*B*—H2*B*⋯O5*B* ^vii^	0.84	1.85	2.663 (6)	163
O3*B*—H3*B*⋯O1*W*	0.84	1.88	2.703 (6)	166
O3*A*—H3*A*⋯O4^iv^	0.84	1.89	2.705 (5)	163
O1*W*—H1*W*1⋯O5*A* ^viii^	0.85 (2)	1.95 (2)	2.794 (7)	173 (8)
O1*W*—H1*W*2⋯O2*B* ^ix^	0.86 (2)	2.16 (5)	2.967 (6)	157 (11)

Symmetry codes: (i) 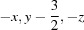; (ii) 
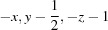
; (iii) 
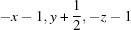
; (iv) 
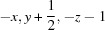
; (v) 
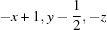
; (vi) 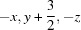; (vii) 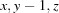; (viii) 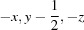; (ix) 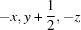.

**Fig. 1.**
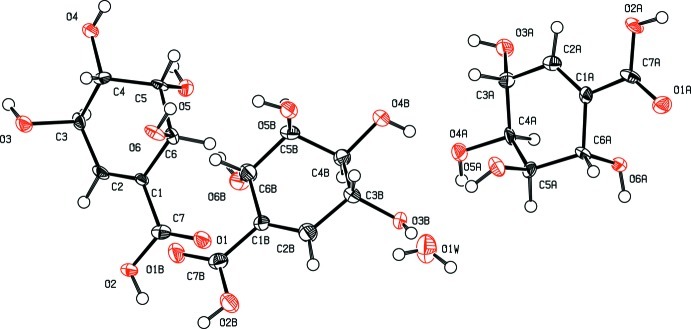
A view of the title compound with the atom numbering scheme. Displacement ellipsoids are drawn at the 50% probability level. H atoms are presented as a small spheres of arbitrary radius.

**Fig. 2.**
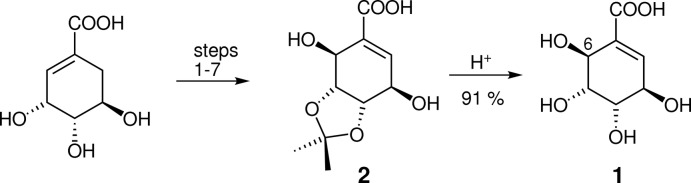
Synthesis of the title compound

**Fig. 3.**
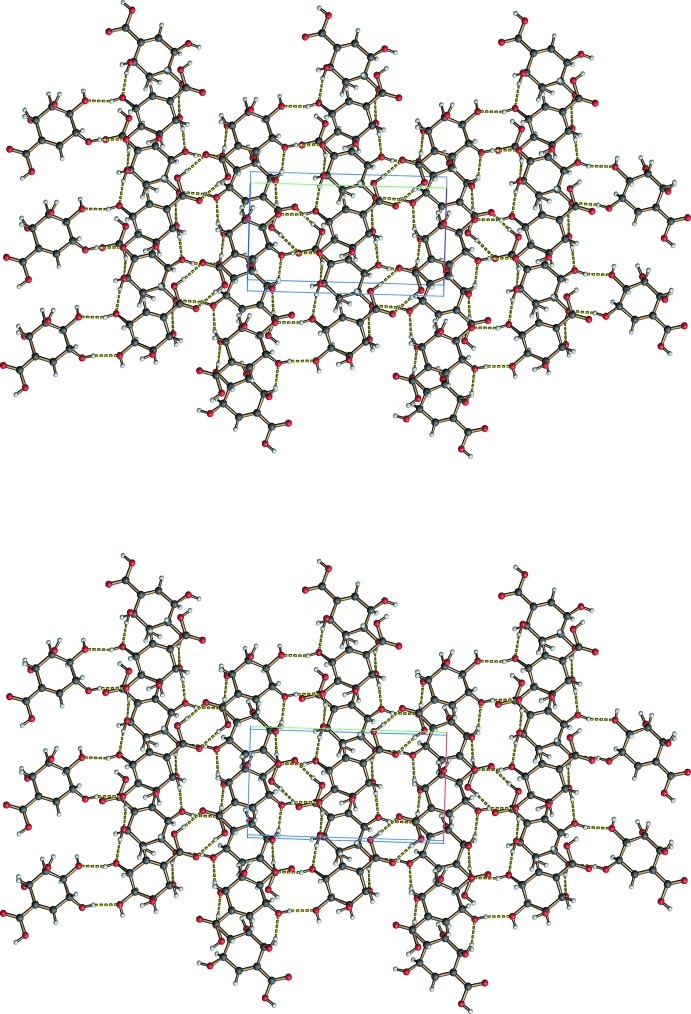
Double layer formed from two independent acid molecules (stereodrawing parallel view).

**Fig. 4.**
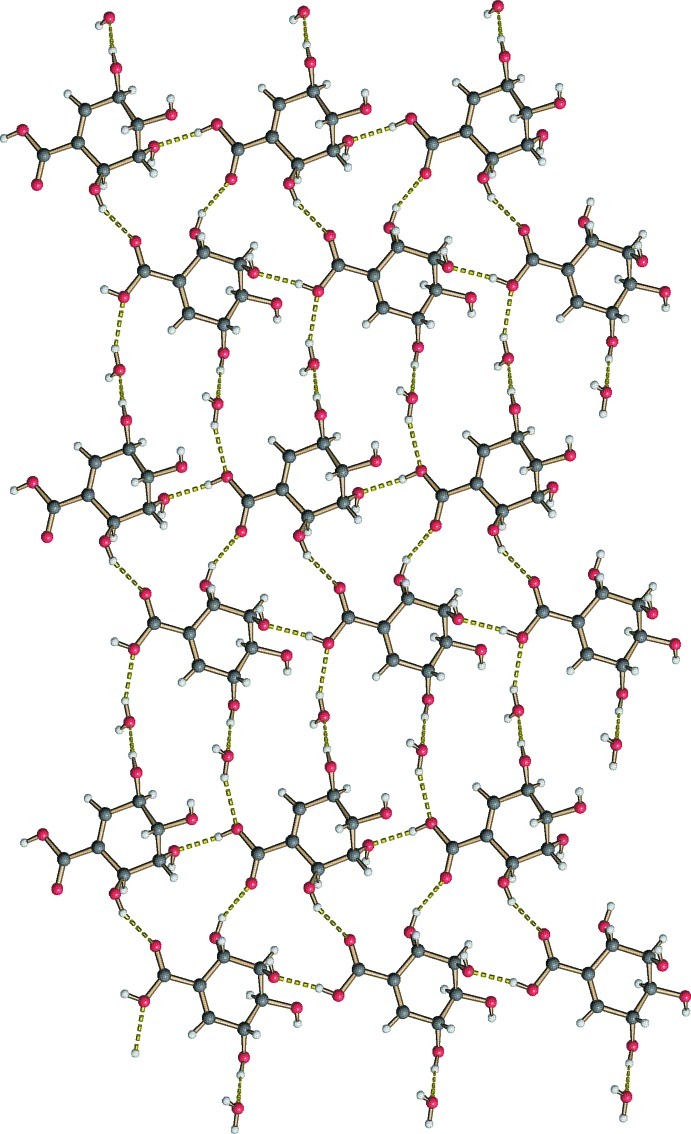
Layer formed from one independent molecule acid and water.

**Fig. 5.**
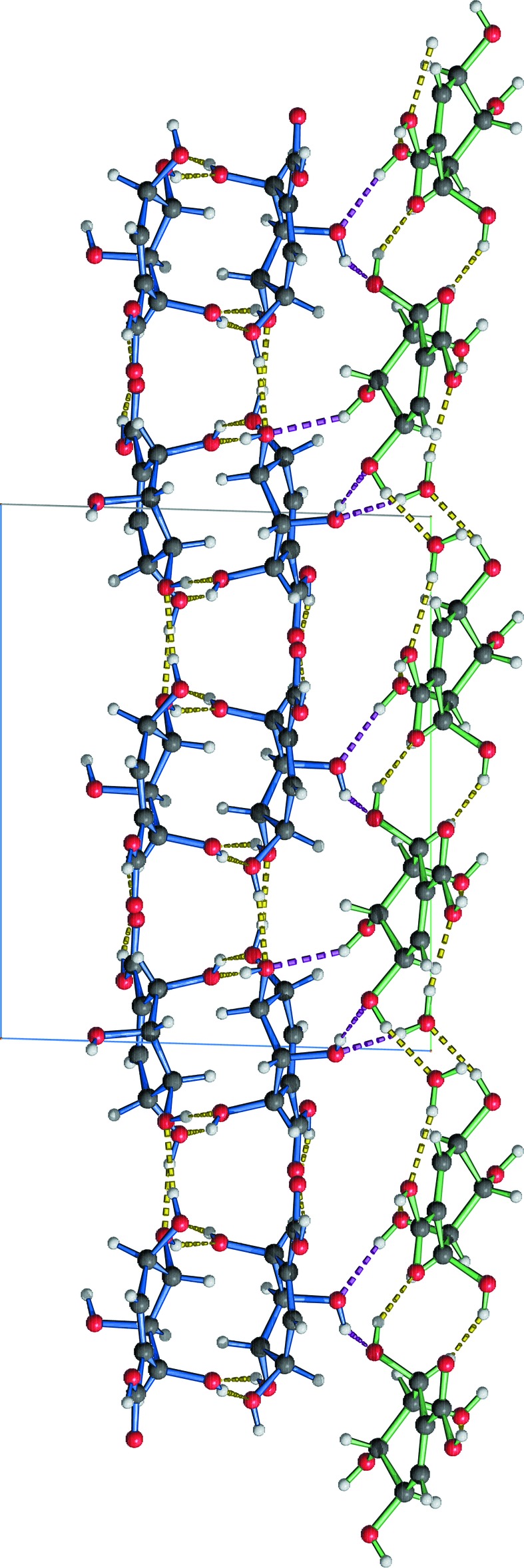
Two connected Layers.

## supplementary crystallographic information

### Comment

The title compound is a enantiomerically pure, highly substituted polyhydroxylated cyclohexane derived from the natural shikimic acid. Polyfunctionalized shikimic acid derivatives, *e.g.* the drug oseltamivir (tamiflu) lately have become well known as drugs repressing the symptoms of bird flu. Furthermore, the 4-epi-shikimic acid skeleton is present in numerous natural products with interesting biological properties, one example is the (6*S*)-6-chloro derivative (pericosine A), an antitumour agent from Periconia byssoid (Usami *et al.*, 2006). Synthetic efforts to new and efficient structural modifications of the shikimate skeleton are thus of current and high relevance.

The asymmetric unit contains three molecules of the acid and one molecule water. All three independent acid molecules have the same half chair conformation (Fig. 1). Two molecules of the acid form hydrogen bonded carboxylic acid dimers, which are connected to double layers by hydrogen bonds (Fig. 3). The third molecule of the acid and the water molecule form a second layer structure (Fig. 4). These two different layer structures are connected *via* additional hydrogen bonds, forming a three dimmensional network (Fig. 5).

### Experimental

By means of a 7-step synthetic procedure (Fig. 2), the acetal 2 was synthesized starting from enantiomerically pure shikimic acid (Griesbeck *et al.*, 2007) by a sequence of 1) esterification (methanol, camphorsulfonic acid), 2) acetalization (dimethoxypropane, camphorsulfonic acid), 3) trifluoromethanesulfonate formation (trifluoromethanesulfonic anhydride, pyridine), 4) dehydration (caesium carbonate, dimethylformamide), 5) singlet oxygenation (rose bengal, visible light, oxygen atmosphere, tetrachloromethane), 6) reduction (potassium iodide, water-acetic acid), and 7) saponification of the methyl ester(lithium hydroxide, water). The acetal 2 was hydrolyzed by the following procedure: To a solution of 60 mg (0.26 mmol) of 2 in 2.5 ml of water and 2.5 ml of methanol was added 2 drops of concentrated HCl under vigorous stirring at room temperature. The reaction mixture was stirred overnight and the solvent evaporated under reduced pressure. The residue was repeatedly dissolved in ethanol and the solvent evaporated to give 45 mg (91%) of the title compound 1 as a colorless product. Recrystallization from ethanol resulted in fine colorless needles, m.p. 140–141°C.

### Refinement

Crystals of 1 are monoclinic; space group *P*2_1_ was chosen as the acid component used was enantiopure sikimic acid and the absolute structure was set by reference to the known chirality of the enantiopure acid employed.

The hydrogen atoms of the hydroxy groups and the water molecule are partially disordered. Only one possible orientation was refined. The positions are constrained and treated as riding atoms with distances O—H = 0.84 Å. All other hydrogen atoms were placed in geometrically idealized positions and refined with using riding model with C—H = 1.00 Å and *U*_iso_(H) = 1.2*U*_eq_(C) for CH, C—H = 0.95 Å and *U*_iso_(H) = 1.2*U*_eq_(C) for C=CH.

### Crystal data

**Table:**

3C_7_H_10_O_6_·H_2_O	*F*(000) = 620
*M**_r_* = 588.47	*D*_x_ = 1.614 Mg m^−^^3^
Monoclinic, *P*2_1_	Mo *K*α radiation, λ = 0.71073 Å
Hall symbol: P 2yb	Cell parameters from 5629 reflections
*a* = 11.2561 (17) Å	θ = 2.3–27.0°
*b* = 7.7049 (11) Å	µ = 0.15 mm^−^^1^
*c* = 13.9688 (14) Å	*T* = 100 K
β = 91.672 (8)°	Prism, colourless
*V* = 1211.0 (3) Å^3^	0.20 × 0.10 × 0.05 mm
*Z* = 2	

### Data collection

**Table:**

Nonius KappaCCD diffractometer	1399 reflections with *I* > 2σ(*I*)
Radiation source: fine-focus sealed tube	*R*_int_ = 0.085
Graphite monochromator	θ_max_ = 27.0°, θ_min_ = 2.3°
φ and ω scans	*h* = −6→14
5629 measured reflections	*k* = −8→9
2786 independent reflections	*l* = −14→17

### Refinement

**Table:**

Refinement on *F*^2^	Primary atom site location: structure-invariant direct methods
Least-squares matrix: full	Secondary atom site location: difference Fourier map
*R*[*F*^2^ > 2σ(*F*^2^)] = 0.054	Hydrogen site location: inferred from neighbouring sites
*wR*(*F*^2^) = 0.102	H atoms treated by a mixture of independent and constrained refinement
*S* = 0.88	*w* = 1/[σ^2^(*F*_o_^2^) + (0.0326*P*)^2^] where *P* = (*F*_o_^2^ + 2*F*_c_^2^)/3
2786 reflections	(Δ/σ)_max_ < 0.001
371 parameters	Δρ_max_ = 0.27 e Å^−^^3^
3 restraints	Δρ_min_ = −0.28 e Å^−^^3^

### Special details

**Table:**

Geometry. All e.s.d.'s (except the e.s.d. in the dihedral angle between two l.s. planes) are estimated using the full covariance matrix. The cell e.s.d.'s are taken into account individually in the estimation of e.s.d.'s in distances, angles and torsion angles; correlations between e.s.d.'s in cell parameters are only used when they are defined by crystal symmetry. An approximate (isotropic) treatment of cell e.s.d.'s is used for estimating e.s.d.'s involving l.s. planes.
Refinement. Refinement of *F*^2^ against all reflections. The weighted *R*-factor *wR* and goodness of fit *S* are based on *F*^2^, conventional *R*-factors *R* are based on *F*, with *F* set to zero for negative *F*^2^. The threshold expression of *F*^2^ > σ(*F*^2^) is used only for calculating *R*-factors(gt) *etc*. and is not relevant to the choice of reflections for refinement. *R*-factors based on *F*^2^ are statistically about twice as large as those based on *F*, and *R*- factors based on all data will be even larger.

### Fractional atomic coordinates and isotropic or equivalent isotropic displacement parameters (Å^2^)

**Table:**

	*x*	*y*	*z*	*U*_iso_*/*U*_eq_	
O1	−0.3102 (4)	−0.2913 (5)	−0.2574 (2)	0.0246 (12)	
O2	−0.3049 (4)	−0.4950 (5)	−0.3725 (2)	0.0237 (11)	
H2	−0.2943	−0.5608	−0.3251	0.036*	
O3	−0.4164 (4)	−0.2120 (5)	−0.6636 (2)	0.0235 (11)	
H3	−0.4002	−0.1782	−0.7189	0.035*	
O4	−0.3797 (4)	0.1684 (5)	−0.6388 (2)	0.0211 (11)	
H4	−0.4069	0.2653	−0.6226	0.032*	
O5	−0.2179 (4)	0.1329 (5)	−0.4720 (2)	0.0199 (11)	
H5	−0.1998	0.1762	−0.5249	0.030*	
O6	−0.4911 (4)	−0.0313 (5)	−0.3704 (2)	0.0226 (11)	
H6	−0.5149	0.0667	−0.3527	0.034*	
C1	−0.3322 (5)	−0.2021 (8)	−0.4214 (4)	0.0133 (14)	
C2	−0.3220 (5)	−0.2449 (8)	−0.5113 (4)	0.0181 (16)	
H2AA	−0.3016	−0.3617	−0.5251	0.022*	
C3	−0.3400 (6)	−0.1242 (8)	−0.5937 (4)	0.0170 (15)	
H3AA	−0.2616	−0.0992	−0.6227	0.020*	
C4	−0.3972 (6)	0.0455 (8)	−0.5639 (4)	0.0195 (16)	
H4AA	−0.4844	0.0266	−0.5567	0.023*	
C5	−0.3437 (6)	0.1095 (8)	−0.4700 (4)	0.0167 (15)	
H5AA	−0.3817	0.2224	−0.4532	0.020*	
C6	−0.3652 (5)	−0.0227 (8)	−0.3891 (4)	0.0166 (15)	
H6AA	−0.3188	0.0098	−0.3297	0.020*	
C7	−0.3140 (5)	−0.3311 (8)	−0.3425 (4)	0.0175 (15)	
O1A	0.3146 (4)	0.7830 (5)	0.2305 (3)	0.0250 (12)	
O2A	0.2868 (4)	0.9807 (5)	0.1148 (2)	0.0272 (12)	
H2A	0.2832	1.0478	0.1620	0.041*	
O3A	0.4144 (4)	0.7044 (5)	−0.1699 (2)	0.0261 (12)	
H3A	0.3921	0.6804	−0.2262	0.039*	
O4A	0.3839 (4)	0.3234 (5)	−0.1463 (2)	0.0213 (11)	
H4A	0.4305	0.2398	−0.1364	0.032*	
O5A	0.2219 (4)	0.3483 (5)	0.0099 (2)	0.0235 (11)	
H5A	0.2113	0.2508	−0.0159	0.035*	
O6A	0.4887 (4)	0.5260 (5)	0.1274 (2)	0.0221 (11)	
H6A	0.5103	0.4374	0.1584	0.033*	
C1A	0.3275 (5)	0.6941 (8)	0.0687 (4)	0.0159 (15)	
C2A	0.3199 (5)	0.7380 (8)	−0.0230 (4)	0.0186 (16)	
H2A1	0.2996	0.8547	−0.0383	0.022*	
C3A	0.3412 (6)	0.6154 (8)	−0.1043 (4)	0.0189 (16)	
H3A1	0.2633	0.5876	−0.1371	0.023*	
C4A	0.3988 (6)	0.4488 (7)	−0.0705 (3)	0.0175 (16)	
H4A1	0.4857	0.4701	−0.0591	0.021*	
C5A	0.3472 (6)	0.3813 (8)	0.0213 (3)	0.0186 (16)	
H5A1	0.3884	0.2709	0.0399	0.022*	
C6A	0.3646 (6)	0.5122 (7)	0.1018 (4)	0.0172 (15)	
H6A1	0.3184	0.4767	0.1586	0.021*	
C7A	0.3083 (6)	0.8202 (8)	0.1454 (4)	0.0187 (16)	
O1B	−0.0345 (4)	−0.3888 (5)	−0.4150 (3)	0.0299 (12)	
O2B	−0.0513 (4)	−0.4555 (5)	−0.2592 (3)	0.0285 (12)	
H2B	−0.0715	−0.5529	−0.2813	0.043*	
O3B	0.1426 (4)	0.0421 (5)	−0.0919 (2)	0.0225 (11)	
H3B	0.1045	0.0246	−0.0419	0.034*	
O4B	0.1501 (4)	0.3201 (5)	−0.2274 (2)	0.0251 (11)	
H4B	0.2020	0.3209	−0.1830	0.038*	
O5B	−0.0695 (4)	0.2162 (5)	−0.3176 (2)	0.0231 (11)	
H5B	−0.1197	0.1992	−0.3623	0.035*	
O6B	0.1309 (4)	−0.0858 (5)	−0.4331 (2)	0.0246 (12)	
H6B	0.1225	−0.0478	−0.4893	0.037*	
C1B	0.0130 (6)	−0.1703 (8)	−0.2996 (4)	0.0208 (16)	
C2B	0.0253 (6)	−0.1288 (8)	−0.2070 (4)	0.0265 (18)	
H2B1	0.0105	−0.2157	−0.1606	0.032*	
C3B	0.0617 (6)	0.0501 (8)	−0.1727 (4)	0.0238 (17)	
H3B1	−0.0107	0.1167	−0.1549	0.029*	
C4B	0.1225 (6)	0.1430 (8)	−0.2535 (4)	0.0227 (17)	
H4B1	0.1979	0.0807	−0.2679	0.027*	
C5B	0.0418 (6)	0.1435 (8)	−0.3425 (4)	0.0198 (16)	
H5B1	0.0782	0.2171	−0.3928	0.024*	
C6B	0.0283 (6)	−0.0423 (8)	−0.3797 (4)	0.0242 (17)	
H6B1	−0.0434	−0.0479	−0.4236	0.029*	
C7B	−0.0249 (6)	−0.3495 (8)	−0.3313 (4)	0.0224 (17)	
O1W	−0.0108 (6)	0.0088 (8)	0.0523 (4)	0.0444 (14)	
H1W1	−0.071 (5)	−0.047 (10)	0.031 (5)	0.08 (4)*	
H1W2	−0.003 (9)	−0.010 (15)	0.113 (2)	0.15 (5)*	

### Atomic displacement parameters (Å^2^)

**Table:**

	*U*^11^	*U*^22^	*U*^33^	*U*^12^	*U*^13^	*U*^23^
O1	0.039 (3)	0.018 (3)	0.017 (2)	−0.003 (2)	0.004 (2)	−0.001 (2)
O2	0.040 (3)	0.012 (3)	0.019 (2)	0.000 (2)	−0.0037 (19)	0.0025 (19)
O3	0.039 (3)	0.016 (3)	0.016 (2)	−0.006 (2)	0.002 (2)	0.0002 (19)
O4	0.034 (3)	0.013 (3)	0.016 (2)	−0.001 (2)	0.0000 (19)	−0.0002 (19)
O5	0.021 (3)	0.020 (3)	0.019 (2)	−0.003 (2)	−0.0026 (19)	0.0009 (18)
O6	0.026 (3)	0.010 (3)	0.032 (2)	−0.003 (2)	0.006 (2)	0.000 (2)
C1	0.013 (4)	0.008 (4)	0.019 (3)	0.001 (3)	−0.003 (3)	−0.001 (3)
C2	0.017 (4)	0.009 (4)	0.028 (4)	−0.006 (3)	−0.001 (3)	0.001 (3)
C3	0.014 (4)	0.016 (4)	0.021 (3)	−0.005 (3)	−0.003 (3)	−0.004 (3)
C4	0.025 (4)	0.015 (4)	0.018 (3)	0.003 (3)	−0.002 (3)	0.006 (3)
C5	0.022 (5)	0.006 (3)	0.022 (3)	−0.003 (3)	0.002 (3)	0.003 (3)
C6	0.016 (4)	0.015 (4)	0.018 (3)	−0.004 (3)	0.000 (3)	−0.003 (3)
C7	0.007 (4)	0.017 (4)	0.029 (4)	0.003 (3)	0.001 (3)	0.005 (3)
O1A	0.033 (3)	0.023 (3)	0.019 (2)	0.002 (2)	0.002 (2)	0.002 (2)
O2A	0.049 (4)	0.011 (3)	0.022 (2)	0.005 (2)	0.000 (2)	−0.006 (2)
O3A	0.039 (3)	0.021 (3)	0.019 (2)	−0.008 (2)	0.000 (2)	0.002 (2)
O4A	0.024 (3)	0.020 (3)	0.020 (2)	0.002 (2)	−0.0007 (19)	−0.0047 (19)
O5A	0.022 (3)	0.019 (3)	0.029 (2)	−0.002 (2)	−0.001 (2)	−0.0075 (19)
O6A	0.027 (3)	0.013 (3)	0.026 (2)	−0.003 (2)	−0.0100 (19)	0.0020 (19)
C1A	0.013 (4)	0.019 (4)	0.016 (3)	−0.009 (3)	−0.002 (3)	0.001 (3)
C2A	0.016 (4)	0.016 (4)	0.024 (3)	0.001 (3)	0.002 (3)	0.003 (3)
C3A	0.021 (4)	0.018 (4)	0.018 (3)	−0.002 (3)	0.000 (3)	0.002 (3)
C4A	0.023 (5)	0.016 (4)	0.013 (3)	−0.002 (3)	−0.001 (3)	−0.008 (3)
C5A	0.016 (4)	0.019 (4)	0.020 (3)	0.005 (3)	−0.009 (3)	−0.002 (3)
C6A	0.023 (4)	0.008 (4)	0.021 (3)	−0.002 (3)	0.000 (3)	0.000 (3)
C7A	0.014 (4)	0.010 (4)	0.033 (4)	−0.002 (3)	0.006 (3)	0.002 (3)
O1B	0.047 (4)	0.023 (3)	0.020 (2)	−0.006 (2)	−0.001 (2)	−0.004 (2)
O2B	0.035 (3)	0.021 (3)	0.029 (2)	−0.004 (2)	0.001 (2)	−0.004 (2)
O3B	0.030 (3)	0.020 (3)	0.017 (2)	−0.003 (2)	−0.0082 (19)	−0.0005 (18)
O4B	0.035 (3)	0.016 (3)	0.024 (2)	−0.002 (2)	−0.0052 (19)	−0.001 (2)
O5B	0.024 (3)	0.018 (3)	0.027 (2)	0.000 (2)	−0.003 (2)	−0.001 (2)
O6B	0.030 (3)	0.024 (3)	0.021 (2)	0.002 (2)	0.007 (2)	0.0008 (19)
C1B	0.030 (5)	0.016 (4)	0.017 (3)	0.003 (3)	−0.003 (3)	0.000 (3)
C2B	0.034 (5)	0.023 (4)	0.022 (3)	−0.003 (4)	0.002 (3)	0.000 (3)
C3B	0.033 (5)	0.015 (4)	0.023 (3)	0.000 (3)	−0.004 (3)	−0.001 (3)
C4B	0.031 (5)	0.019 (4)	0.018 (3)	0.004 (3)	−0.001 (3)	−0.002 (3)
C5B	0.017 (4)	0.015 (4)	0.027 (3)	0.002 (3)	0.002 (3)	0.004 (3)
C6B	0.028 (5)	0.021 (4)	0.023 (3)	−0.003 (3)	0.001 (3)	−0.001 (3)
C7B	0.021 (5)	0.021 (4)	0.026 (4)	0.002 (3)	−0.001 (3)	0.004 (3)
O1W	0.048 (4)	0.053 (4)	0.032 (3)	−0.011 (3)	−0.001 (3)	−0.008 (3)

### Geometric parameters (Å, º)

**Table:**

O1—C7	1.226 (6)	C1A—C6A	1.530 (8)
O2—C7	1.336 (7)	C2A—C3A	1.502 (8)
O2—H2	0.8400	C2A—H2A1	0.9500
O3—C3	1.449 (7)	C3A—C4A	1.508 (8)
O3—H3	0.8400	C3A—H3A1	1.0000
O4—C4	1.429 (6)	C4A—C5A	1.515 (7)
O4—H4	0.8400	C4A—H4A1	1.0000
O5—C5	1.429 (7)	C5A—C6A	1.518 (7)
O5—H5	0.8400	C5A—H5A1	1.0000
O6—C6	1.450 (6)	C6A—H6A1	1.0000
O6—H6	0.8400	O1B—C7B	1.210 (6)
C1—C2	1.307 (7)	O2B—C7B	1.337 (6)
C1—C7	1.494 (8)	O2B—H2B	0.8400
C1—C6	1.504 (8)	O3B—C3B	1.431 (7)
C2—C3	1.490 (8)	O3B—H3B	0.8400
C2—H2AA	0.9500	O4B—C4B	1.444 (7)
C3—C4	1.521 (8)	O4B—H4B	0.8400
C3—H3AA	1.0000	O5B—C5B	1.425 (7)
C4—C5	1.510 (7)	O5B—H5B	0.8400
C4—H4AA	1.0000	O6B—C6B	1.433 (6)
C5—C6	1.546 (7)	O6B—H6B	0.8400
C5—H5AA	1.0000	C1B—C2B	1.336 (7)
C6—H6AA	1.0000	C1B—C6B	1.506 (7)
O1A—C7A	1.223 (6)	C1B—C7B	1.507 (9)
O2A—C7A	1.329 (7)	C2B—C3B	1.512 (8)
O2A—H2A	0.8400	C2B—H2B1	0.9500
O3A—C3A	1.426 (6)	C3B—C4B	1.516 (8)
O3A—H3A	0.8400	C3B—H3B1	1.0000
O4A—C4A	1.440 (6)	C4B—C5B	1.519 (8)
O4A—H4A	0.8400	C4B—H4B1	1.0000
O5A—C5A	1.437 (7)	C5B—C6B	1.529 (8)
O5A—H5A	0.8400	C5B—H5B1	1.0000
O6A—C6A	1.436 (6)	C6B—H6B1	1.0000
O6A—H6A	0.8400	O1W—H1W1	0.85 (2)
C1A—C2A	1.324 (7)	O1W—H1W2	0.86 (2)
C1A—C7A	1.467 (8)		
			
C7—O2—H2	109.5	O4A—C4A—H4A1	108.6
C3—O3—H3	109.5	C3A—C4A—H4A1	108.6
C4—O4—H4	109.5	C5A—C4A—H4A1	108.6
C5—O5—H5	109.5	O5A—C5A—C4A	111.5 (4)
C6—O6—H6	109.5	O5A—C5A—C6A	107.8 (5)
C2—C1—C7	121.8 (5)	C4A—C5A—C6A	110.7 (5)
C2—C1—C6	123.4 (5)	O5A—C5A—H5A1	108.9
C7—C1—C6	114.8 (5)	C4A—C5A—H5A1	108.9
C1—C2—C3	124.9 (6)	C6A—C5A—H5A1	108.9
C1—C2—H2AA	117.6	O6A—C6A—C5A	109.7 (5)
C3—C2—H2AA	117.6	O6A—C6A—C1A	105.3 (5)
O3—C3—C2	107.1 (5)	C5A—C6A—C1A	110.9 (4)
O3—C3—C4	109.7 (5)	O6A—C6A—H6A1	110.3
C2—C3—C4	112.0 (4)	C5A—C6A—H6A1	110.3
O3—C3—H3AA	109.3	C1A—C6A—H6A1	110.3
C2—C3—H3AA	109.3	O1A—C7A—O2A	122.4 (5)
C4—C3—H3AA	109.3	O1A—C7A—C1A	123.3 (6)
O4—C4—C5	111.1 (5)	O2A—C7A—C1A	114.3 (5)
O4—C4—C3	107.5 (4)	C7B—O2B—H2B	109.5
C5—C4—C3	110.9 (5)	C3B—O3B—H3B	109.5
O4—C4—H4AA	109.1	C4B—O4B—H4B	109.5
C5—C4—H4AA	109.1	C5B—O5B—H5B	109.5
C3—C4—H4AA	109.1	C6B—O6B—H6B	109.5
O5—C5—C4	113.2 (4)	C2B—C1B—C6B	123.5 (6)
O5—C5—C6	105.9 (5)	C2B—C1B—C7B	121.6 (6)
C4—C5—C6	110.7 (5)	C6B—C1B—C7B	114.8 (5)
O5—C5—H5AA	109.0	C1B—C2B—C3B	123.0 (6)
C4—C5—H5AA	109.0	C1B—C2B—H2B1	118.5
C6—C5—H5AA	109.0	C3B—C2B—H2B1	118.5
O6—C6—C1	105.3 (5)	O3B—C3B—C2B	111.8 (5)
O6—C6—C5	109.7 (5)	O3B—C3B—C4B	108.5 (5)
C1—C6—C5	109.9 (4)	C2B—C3B—C4B	108.6 (5)
O6—C6—H6AA	110.6	O3B—C3B—H3B1	109.3
C1—C6—H6AA	110.6	C2B—C3B—H3B1	109.3
C5—C6—H6AA	110.6	C4B—C3B—H3B1	109.3
O1—C7—O2	122.7 (5)	O4B—C4B—C3B	110.9 (4)
O1—C7—C1	123.3 (6)	O4B—C4B—C5B	108.9 (5)
O2—C7—C1	114.0 (5)	C3B—C4B—C5B	109.8 (5)
C7A—O2A—H2A	109.5	O4B—C4B—H4B1	109.1
C3A—O3A—H3A	109.5	C3B—C4B—H4B1	109.1
C4A—O4A—H4A	109.5	C5B—C4B—H4B1	109.1
C5A—O5A—H5A	109.5	O5B—C5B—C4B	108.1 (5)
C6A—O6A—H6A	109.5	O5B—C5B—C6B	111.8 (5)
C2A—C1A—C7A	122.0 (6)	C4B—C5B—C6B	109.1 (5)
C2A—C1A—C6A	122.4 (5)	O5B—C5B—H5B1	109.3
C7A—C1A—C6A	115.5 (5)	C4B—C5B—H5B1	109.3
C1A—C2A—C3A	124.3 (6)	C6B—C5B—H5B1	109.3
C1A—C2A—H2A1	117.9	O6B—C6B—C1B	110.2 (5)
C3A—C2A—H2A1	117.9	O6B—C6B—C5B	108.8 (5)
O3A—C3A—C2A	106.9 (5)	C1B—C6B—C5B	111.9 (4)
O3A—C3A—C4A	111.0 (5)	O6B—C6B—H6B1	108.6
C2A—C3A—C4A	112.0 (5)	C1B—C6B—H6B1	108.6
O3A—C3A—H3A1	108.9	C5B—C6B—H6B1	108.6
C2A—C3A—H3A1	108.9	O1B—C7B—O2B	124.0 (6)
C4A—C3A—H3A1	108.9	O1B—C7B—C1B	122.0 (6)
O4A—C4A—C3A	107.5 (4)	O2B—C7B—C1B	114.0 (5)
O4A—C4A—C5A	110.7 (5)	H1W1—O1W—H1W2	109 (8)
C3A—C4A—C5A	112.6 (5)		
			
C7—C1—C2—C3	179.4 (6)	O5A—C5A—C6A—O6A	169.7 (4)
C6—C1—C2—C3	0.7 (10)	C4A—C5A—C6A—O6A	−68.1 (6)
C1—C2—C3—O3	−132.5 (6)	O5A—C5A—C6A—C1A	−74.4 (6)
C1—C2—C3—C4	−12.2 (9)	C4A—C5A—C6A—C1A	47.7 (7)
O3—C3—C4—O4	−77.6 (6)	C2A—C1A—C6A—O6A	98.2 (6)
C2—C3—C4—O4	163.7 (5)	C7A—C1A—C6A—O6A	−77.9 (6)
O3—C3—C4—C5	160.8 (5)	C2A—C1A—C6A—C5A	−20.4 (8)
C2—C3—C4—C5	42.0 (7)	C7A—C1A—C6A—C5A	163.5 (5)
O4—C4—C5—O5	−62.3 (6)	C2A—C1A—C7A—O1A	179.9 (6)
C3—C4—C5—O5	57.2 (6)	C6A—C1A—C7A—O1A	−4.0 (9)
O4—C4—C5—C6	178.9 (5)	C2A—C1A—C7A—O2A	−2.0 (9)
C3—C4—C5—C6	−61.6 (6)	C6A—C1A—C7A—O2A	174.2 (5)
C2—C1—C6—O6	99.4 (6)	C6B—C1B—C2B—C3B	−3.0 (11)
C7—C1—C6—O6	−79.5 (6)	C7B—C1B—C2B—C3B	−179.2 (6)
C2—C1—C6—C5	−18.7 (9)	C1B—C2B—C3B—O3B	−140.0 (6)
C7—C1—C6—C5	162.5 (5)	C1B—C2B—C3B—C4B	−20.4 (9)
O5—C5—C6—O6	169.9 (4)	O3B—C3B—C4B—O4B	−63.3 (6)
C4—C5—C6—O6	−67.0 (6)	C2B—C3B—C4B—O4B	175.1 (5)
O5—C5—C6—C1	−74.8 (6)	O3B—C3B—C4B—C5B	176.4 (5)
C4—C5—C6—C1	48.3 (7)	C2B—C3B—C4B—C5B	54.7 (7)
C2—C1—C7—O1	171.8 (6)	O4B—C4B—C5B—O5B	−67.1 (6)
C6—C1—C7—O1	−9.3 (9)	C3B—C4B—C5B—O5B	54.4 (6)
C2—C1—C7—O2	−9.7 (9)	O4B—C4B—C5B—C6B	171.1 (5)
C6—C1—C7—O2	169.2 (5)	C3B—C4B—C5B—C6B	−67.4 (6)
C7A—C1A—C2A—C3A	178.6 (6)	C2B—C1B—C6B—O6B	113.1 (7)
C6A—C1A—C2A—C3A	2.7 (10)	C7B—C1B—C6B—O6B	−70.4 (7)
C1A—C2A—C3A—O3A	−134.8 (6)	C2B—C1B—C6B—C5B	−8.1 (10)
C1A—C2A—C3A—C4A	−12.9 (9)	C7B—C1B—C6B—C5B	168.4 (5)
O3A—C3A—C4A—O4A	−77.0 (6)	O5B—C5B—C6B—O6B	160.3 (4)
C2A—C3A—C4A—O4A	163.5 (5)	C4B—C5B—C6B—O6B	−80.2 (6)
O3A—C3A—C4A—C5A	160.8 (5)	O5B—C5B—C6B—C1B	−77.7 (6)
C2A—C3A—C4A—C5A	41.3 (7)	C4B—C5B—C6B—C1B	41.8 (7)
O4A—C4A—C5A—O5A	−60.9 (6)	C2B—C1B—C7B—O1B	−178.8 (6)
C3A—C4A—C5A—O5A	59.4 (6)	C6B—C1B—C7B—O1B	4.6 (10)
O4A—C4A—C5A—C6A	179.1 (5)	C2B—C1B—C7B—O2B	3.9 (10)
C3A—C4A—C5A—C6A	−60.5 (7)	C6B—C1B—C7B—O2B	−172.7 (5)

### Hydrogen-bond geometry (Å, º)

**Table:**

*D*—H···*A*	*D*—H	H···*A*	*D*···*A*	*D*—H···*A*
O2—H2···O1*A*^i^	0.84	1.81	2.624 (5)	164
O3—H3···O4*A*^ii^	0.84	1.90	2.705 (5)	161
O4—H4···O6^iii^	0.84	1.94	2.738 (6)	157
O5—H5···O6*B*^iv^	0.84	2.08	2.736 (5)	134
O6—H6···O3^iii^	0.84	1.89	2.718 (6)	169
O6*A*—H6*A*···O3*A*^v^	0.84	1.99	2.764 (6)	153
O4*A*—H4*A*···O6*A*^v^	0.84	1.88	2.712 (6)	168
O5*B*—H5*B*···O5	0.84	1.93	2.765 (5)	171
O5*A*—H5*A*···O3*B*	0.84	2.06	2.882 (5)	164
O2*A*—H2*A*···O1^vi^	0.84	1.84	2.664 (5)	167
O6*B*—H6*B*···O1*B*^iv^	0.84	2.05	2.801 (5)	149
O4*B*—H4*B*···O4*A*	0.84	2.10	2.835 (6)	147
O2*B*—H2*B*···O5*B*^vii^	0.84	1.85	2.663 (6)	163
O3*B*—H3*B*···O1*W*	0.84	1.88	2.703 (6)	166
O3*A*—H3*A*···O4^iv^	0.84	1.89	2.705 (5)	163
O1*W*—H1*W*1···O5*A*^viii^	0.85 (2)	1.95 (2)	2.794 (7)	173 (8)
O1*W*—H1*W*2···O2*B*^ix^	0.86 (2)	2.16 (5)	2.967 (6)	157 (11)

Symmetry codes: (i) −*x*, *y*−3/2, −*z*; (ii) −*x*, *y*−1/2, −*z*−1; (iii) −*x*−1, *y*+1/2, −*z*−1; (iv) −*x*, *y*+1/2, −*z*−1; (v) −*x*+1, *y*−1/2, −*z*; (vi) −*x*, *y*+3/2, −*z*; (vii) *x*, *y*−1, *z*; (viii) −*x*, *y*−1/2, −*z*; (ix) −*x*, *y*+1/2, −*z*.
